# Silver nanoparticle immunomodulatory potential in absence of direct cytotoxicity in RAW 264.7 macrophages and MPRO 2.1 neutrophils

**DOI:** 10.1080/1547691X.2019.1588928

**Published:** 2019-12

**Authors:** Nasser B. Alsaleh, Valerie C. Minarchick, Ryan P. Mendoza, Bipin Sharma, Ramakrishna Podila, Jared M. Brown

**Affiliations:** a Department of Pharmaceutical Sciences, Colorado Center for Nanomedicine and Nanosafety, Skaggs School of Pharmacy and Pharmaceutical Sciences, University of Colorado Anschutz Medical Campus, Aurora, CO, USA;; b Department of Physics and Astronomy, Laboratory of Nano-biophysics, Clemson University, Clemson, SC, USA

**Keywords:** Nanotoxicology, nanotoxicity, immunomodulation, immunotoxicity, immune activation, suppression

## Abstract

Engineered nanomaterials (ENM) are being used in a wide range of consumer products and pharmaceuticals; hence, there is an increasing risk for human exposure and potential adverse outcomes. The immune system, vital in host defense and protection against environmental agents, is typically initiated and executed by innate effector immune cells including macrophages and neutrophils. Previous literature has reported the immune system as a major target of ENM toxicity; however, there is inconsistency regarding the immunotoxicity of ENM. This could be attributed to differences in ENM physicochemical properties, cellular models examined, biocorona formation, etc. Thus, the current study examined the toxicity and immunomodulatory effects of silver nanoparticles (AgNP), one of the most utilized ENM in consumer and medical products, in two key innate immune cell models, e.g. RAW 264.7 cells (macrophages) and differentiated MPRO 2.1 cells (promyelocytes/neutrophils). The results showed that despite a generation of reactive oxygen species, exposure to 20 nm citrate-coated AgNP was not associated with major oxidative damage, inflammatory responses, nor cytotoxicity. Nevertheless, and most importantly, pre-exposure to the AgNP for 24 h enhanced RAW 264.7 cell phagocytic ability as well as the release of inflammatory cytokine interleukin-6 in response to lipopolysaccharide (LPS). In MPRO 2.1 cells, AgNP pre-exposure also resulted in enhanced phagocytic ability; however, these cells manifest reduced cell degranulation (elastase release) and oxidative burst in response to phorbol myristate acetate (PMA). Taken together, these findings indicated to us that exposure to AgNP, despite not being directly (cyto)toxic to these cells, had the potential to alter immune cell responses. The findings underscore the import of assessing immune cell function post-exposure to ENM beyond the standard endpoints such as oxidative stress and cytotoxicity. In addition, these findings further illustrate the importance of understanding the underlying molecular mechanisms of ENM-cellular interactions, particularly in the immune system.

## Introduction

The field of nanotechnology has introduced novel applications in a broad spectrum of disciplines, including materials science, physics, chemistry, computer science, and biotechnology (Nel et al. 2006, 2009). Today, engineered nanomaterials (ENM) are being produced at a large scale and incorporated into a wide range of consumer products - from electronics to health care products (Buzea et al. 2007; Vance et al. 2015). The medical field has also benefited from nanoscience; a number of promising nanotherapeutics have been developed to significantly improve resolution and targeting and reductions in the levels of off-target effects (Ramos et al. 2017). As a result, increased human and environmental exposure to various ENM is inevitable.

Even with these increases in potential exposures, biological behaviors and any associated adverse effects of many ENMs are largely unknown or poorly studied. A main challenge in studying ENM molecular interactions with mammalian cells is that even a minor alteration in ENM physicochemical properties (e.g. size, shape, surface chemistry, biocorona formation, etc.) or experimental conditions (e.g. preparation of ENM, cell culture conditions, animal models, etc.) can lead to a drastic change in ENM biological behavior (Zhu et al. 2013). Importantly, a growing body of research has demonstrated that the majority of ENM-mediated toxicological outcomes involve direct/indirect interactions with the immune system (Dobrovolskaia and McNeil 2007; Alsaleh and Brown 2018). The immune system is the primary system in host protection against invading pathogens as well as environmental toxicants (e.g. ambient air particulate matter, metals, pesticides, radiation, etc.). These protections are mediated through host innate and adaptive immune responses. Notably, responses of key immune cells including macrophages and neutrophils are critical for the initiation of innate immune responses as well as the development of optimal adaptive immunity (which, in turn, is key in later recruitment of additional innate effector immune cells (including monocytes/macrophages and neutrophils) to eliminate invading pathogens/toxicants; Medzhitov and Janeway 1997). As such, modulating the function of innate effector immune cells as a result of ENM exposures could lead to detrimental consequences for optimal host immune responses (Dobrovolskaia and McNeil 2007; Alsaleh and Brown 2018).

Today, one of the most widely used ENM in consumer and medical products are silver nanoparticles (AgNP; Vance et al. 2015). These are utilized in several applications for many reasons, including their optical and electrical properties; moreover, AgNP is largely used due to their apparent antimicrobial properties. Importantly, despite their wide biodistribution and ability to undergo bioaccumulation, AgNP has been found to be generally inert or induce minor organ toxicity in rodent models (Kim et al. 2008; Park et al. 2011a, 2011b). On the other hand, recent evidence has demonstrated potential immunomodulatory properties for various AgNP (de Jong et al. 2013; Vandebriel et al. 2014). For instance, it was shown that *in vivo* exposure of rats to 20 nm AgNP (intravenously, daily for 28 days) led to increased spleen weight and neutrophil infiltration reduced thymic weights and natural killer (NK) cell activity, and suppression of T-cell dependent antibody production. Those findings suggested that despite a lack of direct tissue or system toxicity, chronic exposure to certain AgNP could be associated with adverse health outcomes.

Previous *in vitro* research has demonstrated direct cytotoxicity of ENM (including some AgNP) on several immune cell models (Yang et al. 2012; Hamilton et al. 2014; Aldossari et al. 2015; Liz et al. 2015; Alsaleh et al. 2016; Vallieres et al. 2016; Muller et al. 2018). Furthermore, emerging evidence has suggested potential immunomodulatory properties of metal and metal-oxide ENM at sub-cytotoxic concentrations which do not result in reduced cell viability (Comfort et al. 2011; Andersson-Willman et al. 2012; Seydoux et al. 2014). Nevertheless, due to differences in AgNP physicochemical properties, choices of cellular models, variations in toxicological/immunological endpoints examined, comparisons between previous studies are almost impossible. Indeed, despite efforts, this has been a challenge in the basic assessment of AgNP safety (Bonner et al. 2013; Xia et al. 2013).

Accordingly, this study investigated cellular responses to AgNP in two key innate effector immune cell models, i.e. a macrophage model (RAW 264.7 cells) and a promyelocyte/neutrophil model (MPRO 2.1 cells). Specifically, the studies here assessed direct cellular toxicities in response to 20 nm AgNP; endpoints evaluated in the cell lines included viability, AgNP uptake, reactive oxygen species (ROS) generation, oxidative stress, and inflammatory responses. These studies also investigated potential changes in cellular function and activation to known immunological stimulants (i.e. 24 h post-exposure to the test AgNP).

## Materials and methods

### Nanoparticle characterization

Hydrodynamic size (nm), zeta (ζ) potential (mV), and polydispersity index (PDI) were measured for 20 nm BioPure™ citrate-coated AgNP (NanoComposix, San Diego, CA) using a Zetasizer (Malvern, Westborough, MA) in DI water (nanoparticle vehicle) and cell culture media (Table 1). Transmission electron microscopy (TEM) was used to confirm the size and shape of AgNP (Supplementary Figure S1).

### Cell culture

RAW 264.7 cell line (TIB-71™) and MPRO 2.1 cell line, Clone 2.1 (CRL-11422™) were purchased from American Type Culture Collection (ATCC, Manassas, VA). Cells were cultured at 37 °C under 5% carbon dioxide (CO_2_) following standard aseptic techniques. RAW 264.7 cells were cultured in Dulbecco’s Modified Eagle’s Medium (DMEM) supplemented with 10% fetal bovine serum (FBS), 100 U penicillin/ml, and 100 μg streptomycin/ml. MPRO 2.1 cell line were cultured in Iscove’s Modified Dulbecco’s Medium (IMDM) supplemented with 20% heat-inactivated horse serum as well as 4 mM L-glutamine, 100 U penicillin/ml, 100 μg streptomycin/ml, and 10 ng murine granulocyte-macrophage colony stimulating factor (GM-CSF)/ml. All culture reagents were purchased from Gibco (Waltham, MA).

### Differentiation of MPRO 2.1 cells

MPRO 2.1 cells were differentiated into neutrophils by treatment with *all*-trans retinoic acid (ATRA; Sigma, St. Louis, MO) as previously described (Gaines et al. 2005). In brief, cells were seeded into 24-well cell culture plates and media was supplemented with ATRA (10 μM; replaced fresh every day for three consecutive days). To confirm cell differentiation, cells were harvested and transferred to slides using a Cytospin 4 system (ThermoFisher Scientific, Waltham, MA) and then stained using HEMA3® stain (similar to Wright-Giemsa stain; Sigma). Cells were then evaluated using an Olympus BX43 (Olympus America Inc., Center Valley, PA) at 40X magnification (Supplementary Figure S2(A)). Cell differentiation was also quantified by measuring the surface expression of CD11b (Supplementary Figure S2(B)) using a BD Accuri C6 flow cytometer (BD Biosciences, San Jose, CA) and associated software. In all cases, a minimum of 10,000 events/sample was acquired.

### Cell viability

Cell viability was assessed using an 3-(4,5-dimethylthiazol-2-yl)-2,5-diphenyltetrazolium bromide (MTS) assay kit (Promega, Madison, WI), according to manufacturer instructions. In brief, cells were seeded into 96-well cell culture plates and grown to 80% confluency. At that point, the cells were exposed to the AgNP (at 6.25, 12.5, 25, 50, or 100 μg/ml) for 1, 6, 12, or 24 h (for each dose). Each dose/time was examined in triplicate. After a given time period, plates were centrifuged at 300 × *g* for 5 min and media was removed and replaced with phenol red-free DMEM/F12 HyClone media (GE, Pittsburgh, PA). Thereafter, MTS reagent was added to each well and the plates were incubated at 37 °C for 20 min until color development. The plates were then centrifuged at 300 × *g* for 5 min and supernatants from each were collected and transferred to new 96-well plates. Absorbance in each well was then measured at 490 nm in a Synergy HT system (BioTek, Winooski, VT). Cells that were treated with hydrogen peroxide (H_2_O_2_; 10 mM) for 60 min was used as a positive control.

### Nanoparticle uptake by cells

AgNP uptake was measured by inductively coupled plasma mass spectrometry (ICP-MS) as described previously (Aldossari et al. 2015). In brief, cells were seeded into 24-well culture plates and grown to 80% confluency. A concentration of 50 μg/ml was utilized for most subsequent experiments because it was not associated with reduced viability and is more relevant to previous literature than would be the higher doses examined in the viability studies. Following five washes with ice-cold phosphate-buffered saline (PBS, pH 7.4) to remove any suspended AgNP that had not been internalized by cells, the cells were pelleted by centrifugation (600 x g, 2 min). The resultant final pellet was dissolved in 1 ml 2% HNO_3_ and AgNP uptake was then quantified using an X Series II ICP-MS system (ThermoFisher Scientific). An internal standard containing lithium (Li), yttrium (Y,) and indium (In) was used throughout. All metals were detected at a level of 0.05 ppb resolution.

### Formation of ROS

Reactive species formation in the cells was measured using dichlorofluores cin diacetate (H_2_DCFDA;ThermoFisher Scientific) staining followed by flow cytometry. In brief, cells were cells were seeded into 24-well culture plates and grown to 80% confluency. At that point, the cells were exposed to the AgNP (at 50 μg/ml) for 0.25, 0.5, 1, 2, 6, or 24 h. At each timepoint, cells were then washed with PBS and covered with PBS containing 5 μM H_2_DCFDA. The cells were then incubated at 37 °C for 30 min (protected from light). The cells were then harvested and total fluorescence was measured using the BD Accuri C6 flow cytometer.

### Quantification of cell protein and lipid oxidation

Protein expression was assessed by Western blot as previously described in Alsaleh et al. (2016). In brief, cells were seeded into 12-well culture plates and grown to 80% confluency. The cells were then exposed to 6.25, 12.5, 25, or 50 μg AgNP/ml for 24 h. Parallel cultures were treated with 25 mμ 4-HNE (Cayman, Ann Arbor, MI) for 1 h (positive control). Following harvest and washings with ice-cold PBS, cell pellets were lysed on ice for 45 min with ice-cold Tris-HCl based lysis buffer containing 1% sodium dodecyl sulfate (SDS), protease (1:100), and phosphatase inhibitors (1:100). Resulting lysates were briefly sonicated and then centrifuged at 15, 000 × *g* at 4 °C for 10 min; final supernatants were collected and protein content was determined using the Bradford method. Aliquots of cell proteins (20 μg) were removed, boiled for 5 min in 5% β-mercaptoethanol-containing 4X Laemmli sample buffer, and loaded into SDS polyacrylamide gels for separation. After resolution, the proteins were electro-blotted onto nitrocellulose membranes (overnight, 10 V, 4 °C). All membranes were blocked with 5% bovine serum albumin (BSA) in Tween 20-containing TBS (TBS-T) for 1 h before being incubated overnight at 4 °C in a solution of 5% BSA/TBS-T containing a 1:1000 dilution of primary mouse anti-4-hydroxy-none-nal monoclonal antibody (4-HNE; R&D Systems, Minneapolis, MN). After repeated washing with TBS-T, the membranes were probed with horseradish peroxidase (HRP)-linked secondary anti-mouse IgG antibody (in 5% BSA/TBS-T) for 1 h at room temperature. The membranes were then washed and developed for signal presence using an enhanced chemiluminescence (ECL) substrate (ThermoFisher Scientific) and a ChemiDoc Imaging System (Bio-Rad, Hercules, CA). Relative densities (relative to β-actin housekeeping protein) were quantified using system-associated ImageLab Software.

For detection of oxidized proteins, an OxiSelect™ protein carbonyl immunoblot kit (Cell BioLabs, San Diego, CA) was employed and manufacturer instructions were followed. In brief, each blotted membrane (containing 20 μg protein lysate/spot) was equilibrated in TBS containing 20% methanol for 5 min and then washed in 2 N HCl for 5 min. Membranes were then derivatized for 5 min with dinitrophenylhydrazine (DNPH; a 1X working solution in 2 N HCl (prepared from 10X kit stock)), then washed three times in 2 N HCl, followed by five washes in 50% methanol-TBS. The membranes were then blocked in 5% nonfat milk in PBS and incubated with kit-provided antibodies as described above.

### Quantification of gene expression

Gene expression was measured based on the amplification of mRNA transcripts using real-time PCR (Applied Biosystems StepOnePlus, ThermoFisher Scientific). In brief, cells were seeded into 24-well culture plates and grown to 80% confluency, after which they were exposed to AgNP at the indicated concentrations and for the noted times. At each timepoint, the plated were centrifuged and well supernatants were discarded. The remaining cell pellets were treated with TRI Reagent (Sigma) and then total RNA was isolated using a Direct-20L™ RNA MiniPrep kit (Zymo Research, Irvine, CA). RNA concentration and quality were assessed using a Nano-Drop 2000 system (ThermoFisher Scientific). Sample mRNA was then reverse transcribed into cDNA using an iScript™ cDNA Synthesis kit (Bio-Rad) and a thermocycler (Eppendorf, Hauppauge, NY). Real-time PCR was performed using a ssoAdvanced™ Universal SYBR Green Supermix (Bio-Rad) and QuantiTect primer sets (Qiagen, Germantown, MD). Gene expression was quantified using the ΔΔC_T_ method, relative to a housekeeping gene (*GAPDH*).

### Quantification of cytokine release by enzyme-linked immunosorbent assay (ELISA)

Cytokine release was measured using a DuoSet® ELISA Development System (R&D Systems, Minneapolis, MN), following manufacturer instructions. In brief, cells were seeded into 24-well culture plates and grown to 80% confluency, after which they were exposed to AgNP (50 μg/ml) for 24 h, LPS alone (1 ng/ml; Type O55:B5 from *Escherichia coli*, Sigma) for 24 h, or AgNP (50 μg/ml) for 24 h followed by LPS (1 ng/ml) for 24 h. At the end of each period, culture supernatants were collected and cytokine content measured after incubating aliquots of each supernatant in capture antibody-coated 96-well plates for 2 h at room temperature. All samples were analyzed in triplicate. Plates were then washed with ice-cold PBS-T (0.05%) and then incubated with a kit-provided HRP-conjugated detection antibody for 2 h at room temperature. After incubation and gently washing to remove unbound antibody, kit 3,3’,5,5’-tetramethylbenzidine (TMB) substrate solution was added to each well and the plate held at room temperature until color development. Reactions were stopped by the addition of 50 μl 2 N H_2_SO_4_ to each well and absorbance was then measured at 405 nm in the Synergy HT system. Serial dilutions of standards were used to permit extrapolation of the content of each cytokine in each supernatant.

### Measurement of glutathione (GSH) levels

Total intracellular GSH (combination of all GSH + GSSG present) levels in the cells were assessed using HPLC as described in Jones and Liang (2009). In brief, cells were plated and after reaching 80% confluency, exposed to AgNP (50 μg/ml) for 24 h. After washing with ice-cold PBS, cells were re-suspended in 500 μl of a solution of 5% perchloric acid, 0.2 M boric acid, and 10 μM γ-glutamyl glutamate (internal standard; Acros Organics; Geel, Belgium). Each sample was briefly sonicated and centrifuged at 15, 000 x *g* for 2 min at 4 °C. An aliquot (300 μl) of the resulting supernatant was transferred to an Eppendorf tube and treated with 60 μl of 9.3 mg/ml iodoacetic acid (pH kept in range of 8.8–9.2) and the sample was then derivatized by the addition of 300 μl of a 20 mg dansyl chloride/ml solution and incubation at room temperature overnight. After the aqueous and organic layers were separated using chloroform, materials from the top layer underwent high-performance liquid chromatography (HPLC) analysis over a Supelcosil (LC-NH_2_ 25-cm × 4.6-mm, 5 μm i.d.) column in an Agilent 1200 series system (Agilent, Santa Clara, CA). Total levels of GSH were calculated relative to an internal standard (γ-glutamyl glutamate) and expressed in terms of μM GSH present. GSH levels were calculated relative to total protein content (measured by Bradford method) in corresponding samples.

### Cell phagocytosis

Cell phagocytic activity was measured based on the uptake of latex beads coated with fluorophore-labeled rabbit IgG, according to manufacturer instructions (Cayman, Ann Arbor, MI). In brief, cells were plated in a 24-well dishes and grown until reaching 80% confluency; at that point, the medium was removed, and cells were then exposed to 50 μg AgNP/ml for 24 h. After this, the cells were washed and treated with latex bead-containing fresh culture media that delivered particles at a ratio of 100:1 cell. The cells were incubated at 37 °C for 4 h, then gently washed with PBS before being collected by centrifugation and resuspension in PBS. Cells were gated to exclude any debris or dead cells; 10,000 events/sample were measured for total fluorescence using the BD Accuri™ C6 a flow cytometer.

### Neutrophil degranulation

Neutrophil degranulation, based on the release of elastase into culture supernatants, was assessed using another DuoSet ELISA Development System (R&D Systems, Inc., Minneapolis, MN). In brief, cells were plated in 24-well dishes and grown until reaching 80% confluency; at that point, the medium was removed and the cells were then exposed to 50 μg AgNP/ml medium for 24 h. After this period, the cells were washed with fresh media and the media containing 1 μg/ml phorbol myristate acetate (PMA, Cayman) or medium only (for basal activity measurements) was then added to the cells. After 1 h, culture well supernatants were collected and neutrophil elastase was then quantified in the same manner as described in the section “Quantification of cytokine release by ELISA”.

### Neutrophil oxidative burst

Neutrophil respiratory burst was assessed based on oxidation of the fluorescent dihydrorhodamine 123 probe (DHR 123; Cayman) as previously described (Sarantis and Gray-Owen 2012). In brief, MPRO 2.1 cells were plated in 24-well dishes and grown until reaching 80% confluency; at that point, the medium was removed and the cells were then exposed to 50 μg AgNP/ml for 24 h. Later the cells were then washed with calcium (Ca^2+^)-free Hanks balanced salt solution (HBSS) and re-suspended in DHR 123 (10 μM)-containing HBSS (with Ca^2+^) for 15 min at 37 °C, protected from any light. The cells were then treated with 1 μg PMA/ml (or medium for basal activity measures) for 15 min at 37 °C, again protected from any light. Cells were then collected, centrifuged/washed, and then the total fluorescence was measured in the BD Accuri™ C6 flow cytometer. Cells were gated to remove any debris or dead cells; 10, 000 events were counted.

### Statistical analysis

Data are presented as mean ± standard error of mean (SEM). A one-way or two-way analysis of variance (ANOVA) with Bonferroni *post-hoc* testing was utilized to test for significant differences between multiple treatment groups. A Student’s *t*-test was used where applicable to test for significant differences between two treatment groups. A *p* value <0.05 was considered statistically significant. Prism 5 software (GraphPad Inc., San Diego, CA) was used for all data analysis and generation of graphs. When values are presented as “relative to control”, this indicates these values were calculated relative to the “mean value of control biological replicates”.

## Results

### Cell viability following exposure to silver nanoparticles (AgNP)

**Figure 1.**
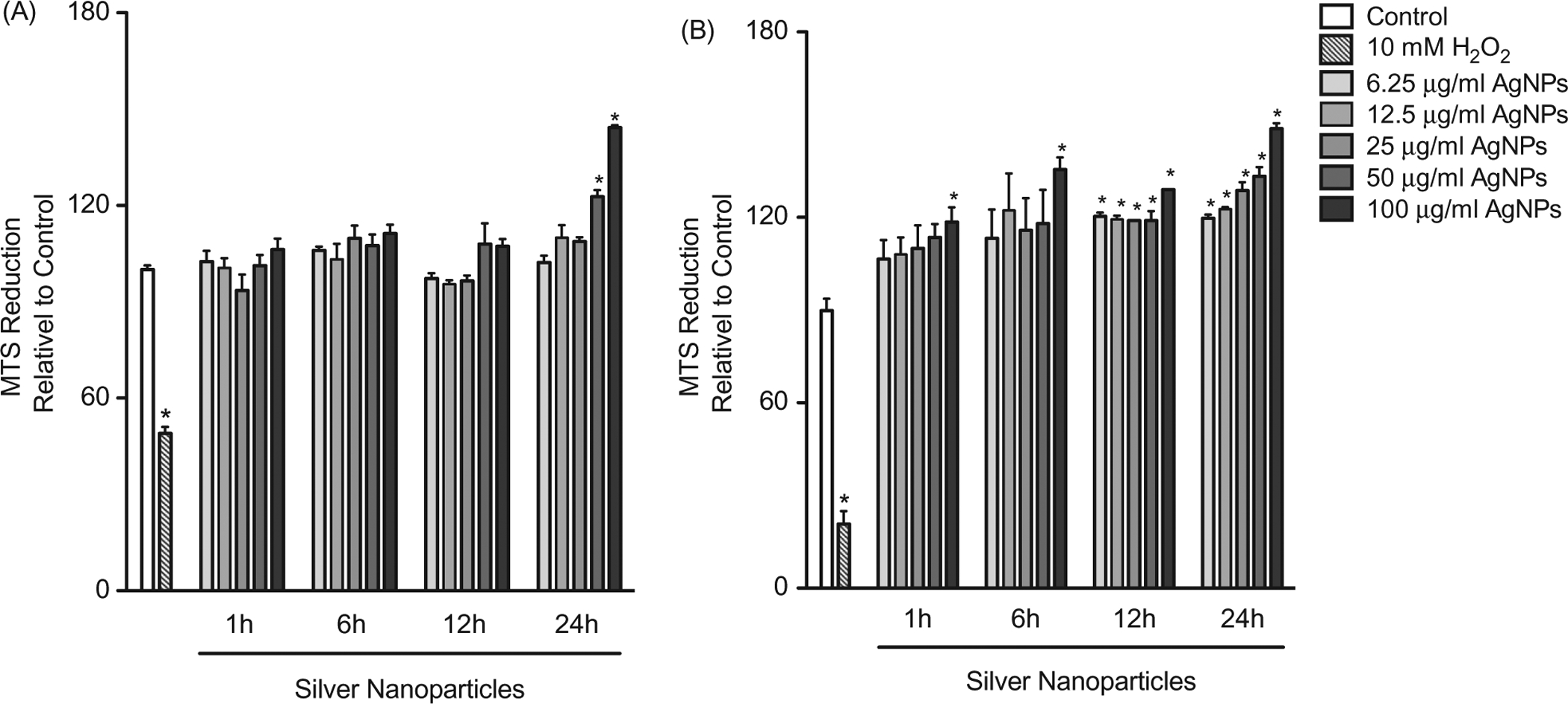
Cell viability following exposure. (A) RAW 264.7 or (B) MPRO 2.1 cells were exposed to silver nanoparticles (AgNP) at 6.25, 12.5, 25, 50, or 100 μg/ml for 1, 6, 12, or 24 h before cell viability was assessed based on viable cell formation of formazan. Treatment of cells with hydrogen peroxide (10 mM) for 60 min was used as a positive control. Values shown are means ± SEM (*N* ≥ 3). *p < 0.05 vs. control group.

To assess cell viability following exposure to the AgNP, cells were exposed to a range of AgNP concentrations (6.25–100 μg/ml) and for multiple timepoints (1, 6, 12, or 24 h). Cell viability was based on the formation of formazan by mitochondrial dehydrogenases (MTS assay). The data showed that exposure to the AgNP was not associated with reduced viability even at high concentrations of the AgNP and over long exposure periods (Figure 1). In fact, at high concentrations and long exposure times, there was an enhanced MTS reduction that was more prominent in the MPRO 2.1 cells (Figure 1).

### Cellular uptake of AgNP

**Figure 2.**
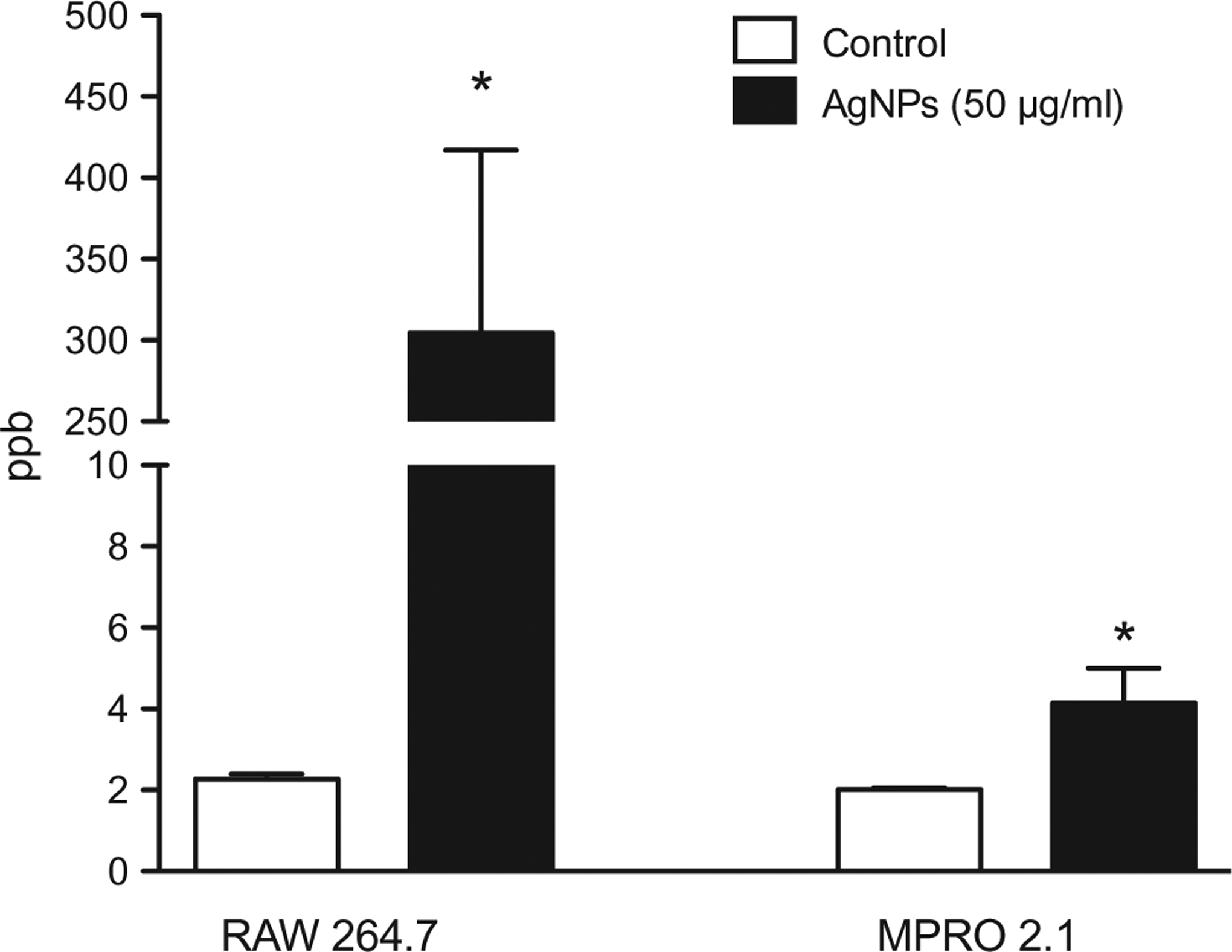
Silver nanoparticle uptake. RAW 264.7 or MPRO 2.1 cells were exposed to the AgNP (50 μg/ml) for 24 h. Particle uptake over the period was then quantified by inductively-coupled plasma mass spectrometry. Values shown are means ± SEM (*N* ≥ 3). *p < 0.05 vs. control.

Assessing the uptake of AgNP by professional phagocytic cell type such as macrophages is key to better understanding ENM cell membrane vs. intracellular interactions. As expected, the results here showed there was significant uptake of AgNP by the RAW 264.7 cells 24 h post-exposure to 50 μg AgNP/ml (Figure 2). However, the data demonstrated that exposure of the MPRO 2.1 cells to the AgNP (50 μg/ml) was associated with a minimal level of uptake.

### ROS formation and oxidative damage following exposure to AgNP

**Figure 3.**
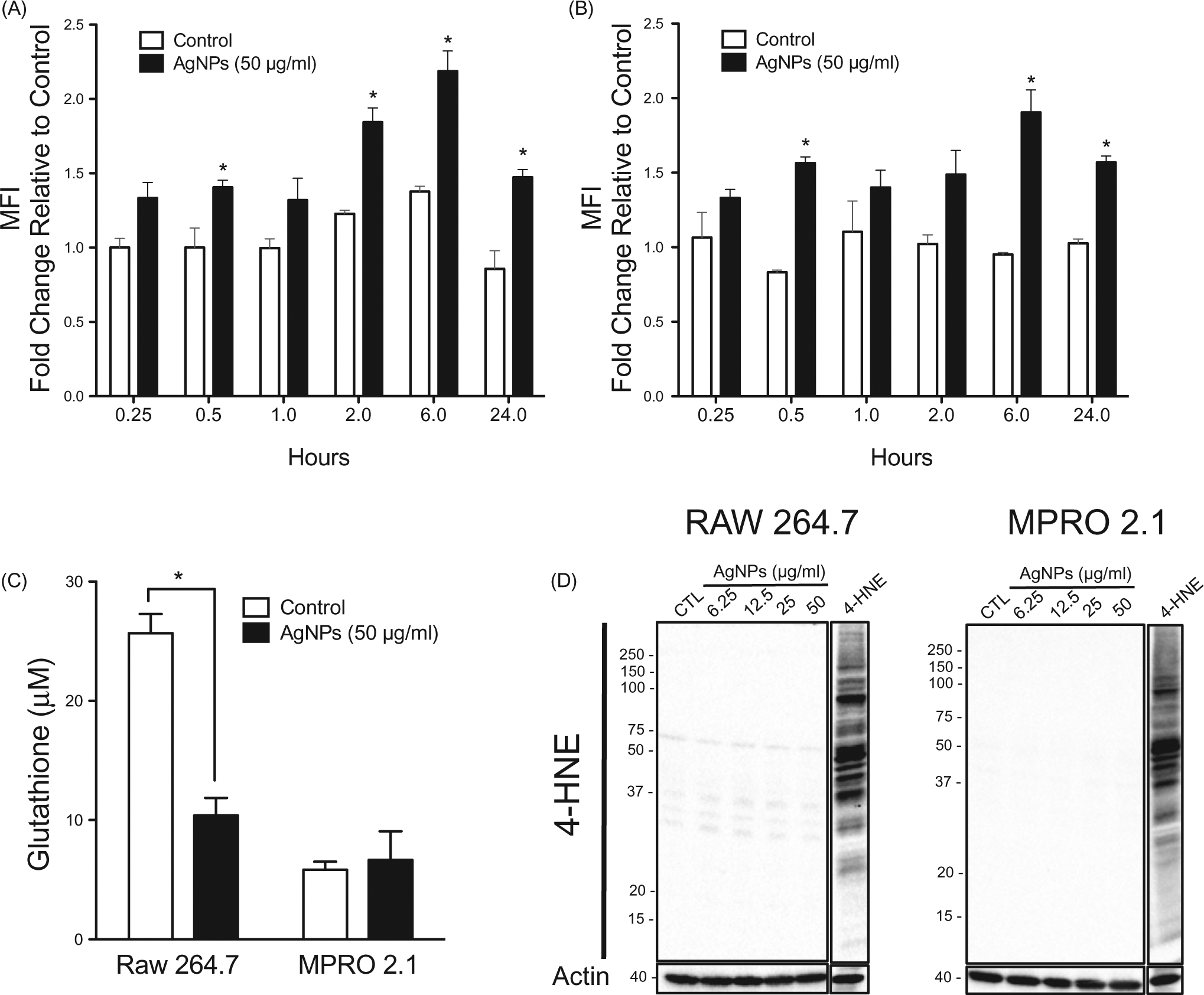
Reactive species generation, glutathione levels, and lipid peroxidation following exposure. (A) RAW 264.7 or (B) MPRO 2.1 cells were exposed to AgNP (50 μg/ml) for 0.25, 0.5, 1, 2, 6, or 24 h. At each timepoint, ROS generation was quantified based on fluorescence of cleaved H_2_DCFDA. (C) RAW 264.7 or MPRO 2.1 cells were exposed to AgNP (50 μg/ml) for 24 h and total glutathione levels were then measured by HPLC. (D) RAW 264.7 or MPRO 2.1 cells were exposed to AgNP (at 6.25, 12.5, 25, or 50 μg/ml) for 24 h and then lipid peroxidation (based on formation of 4-HNE) that had occurred in the cells was assessed by immunoblotting. Cells treated with 4-HNE (25 μM) for 1 h served as a positive control. Values shown are means ± SEM (*N* ≥ 3). *p < 0.05 vs. control.

Due to their large surface to volume ratio, ENM including AgNP are typically associated with the generation of ROS in cellular systems (Fu et al. 2014). The data here indicated that exposure to the AgNP (at 50 μg/ml) triggered ROS generation in both cell types. Specifically, in RAW 264.7 cells, while there was limited ROS generation at the early timepoints (i.e. within 1 h), a burst in ROS production was observed at the late timepoints - peaking at 6 h and subsiding by 24 h of exposure (Figure 3(A)). In MPRO 2.1 cells, a similar pattern of ROS generation was observed that also peaked at 6 h (Figure 3(B)).

To examine whether exposure to the AgNP would result in activation of cell anti-oxidant responses or lead to oxidative stress, gene expression of enzymes involved in cell anti-oxidant systems (i.e. glutathione peroxidase-1 (GPx1) and catalase-1 (Cat1)) as well as levels of total glutathione (GSH + GSSG) were assessed following exposure to the AgNP for 24þh. The data revealed no major changes in *GPx1* and *Cat-1* gene expression following the exposure in both cell lines (Supplementary Figure S3). However, intracellular GSH levels were reduced in RAW 264.7 cells following exposure to the AgNP; there was no impact on GSH levels in the MPRO 2.1 cells (Figure 3(C)). To assess macromolecular oxidation (including lipid peroxidation and protein oxidation) following exposure to the AgNP for 24 h (so as to exclude any irreversible oxidative damage secondary to ROS generation and depletion of GSH), formation of 4-hydroxynonenal (4-HNE; marker of lipid peroxidation) and protein carbonyl derivatives (based on derivatization of carbonyls with dinitrophenylhydrazine, DNPH) as a marker of protein oxidation was assessed. Our data showed that exposure to AgNP (6.25–50 μg/ml) for 24 h was neither associated with increases in the incidence of lipid peroxidation (Figure 3(D)) or protein oxidation (Supplementary Figure S4). Collectively, the current results indicated that exposure to AgNP in the range of 6.25–50 μg/ml for up to 24 h could reduce cellular total GSH levels after ingestion of the particles, but this loss of GSH was not associated with major oxidative damage or cellular toxicity in either cell line.

### Inflammatory response following exposure to silver nanoparticles

**Figure 4.**
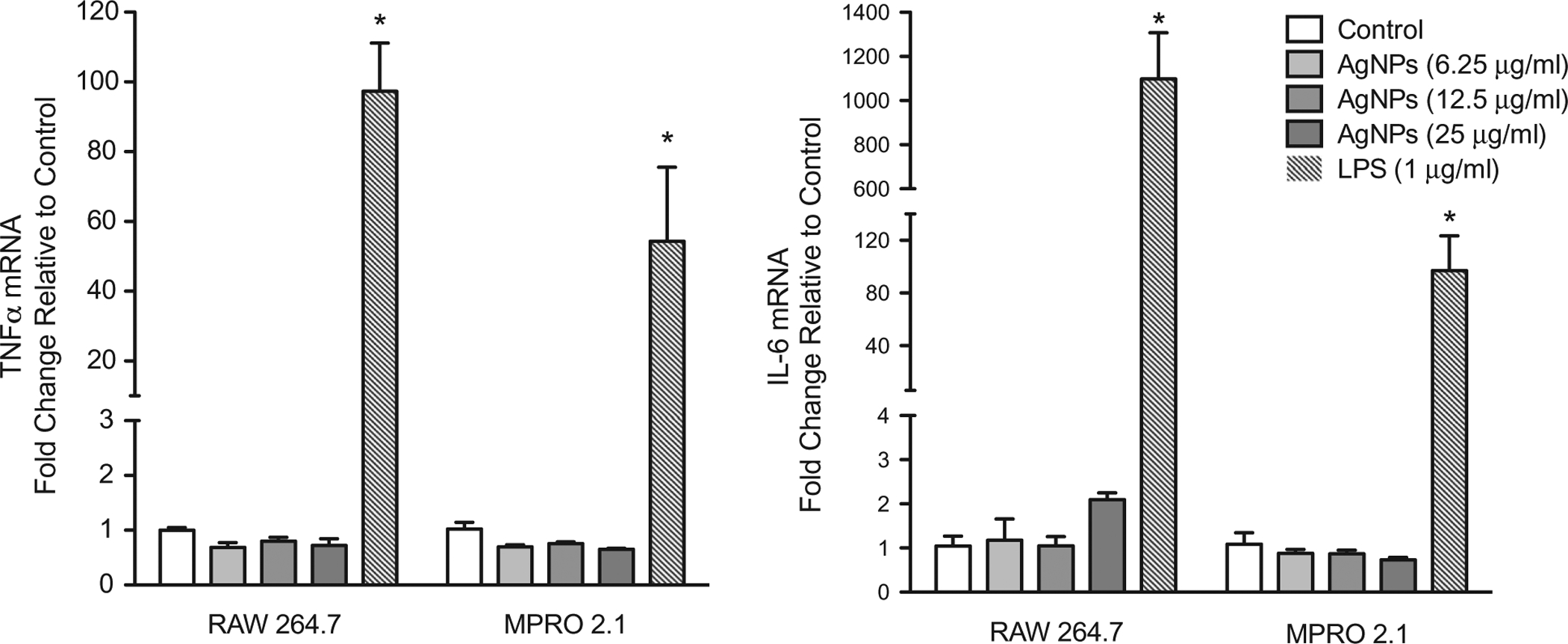
Inflammatory cytokine gene expression following exposure. RAW 264.7 or MPRO 2.1 cells were exposed to the AgNP (at 6.25, 12.5, or 25 μg/ml) for 6 hr. TNFα and IL-6 gene expression in the cells was then measured by real-time PCR. Values shown are means ± SEM (*N* ≥ 3). *p < 0.05 vs. control.

Synthesis and release of cytokines/chemokines by immune cells are key during immune responses. Despite the lack of any overt significant cytotoxicity in the test cell lines due to the AgNP, the current study still sought to assess whether the exposure was potentially associated with an induced inflammatory response. The results showed that exposure to the AgNP for up to 6 h was not associated with up-regulation of tumor necrosis factorα (TNFα) or interleukin-6 (IL-6) mRNA expression levels in either cell line (Figure 4), indicating an absence of a major pro-inflammatory response induced in response to this specific AgNP exposure.

### Cell function following exposure to silver nanoparticles

Since AgNP exposure here was associated with neither major cellular toxicity nor inflammatory responses and because previous literature demonstrated potential immunomodulatory effects of AgNP in the absence of major toxicities, the current study then sought to assess if pre-exposure to the AgNP could influence normal cellular function or activation by known stimuli. Specifically, cell phagocytic activity as well as activation of RAW 264.7 and MPRO 2.1 cells, in response to respectively LPS or PMA, following exposure to the AgNP (50 μg/ml) for 24 h.

**Figure 5.**
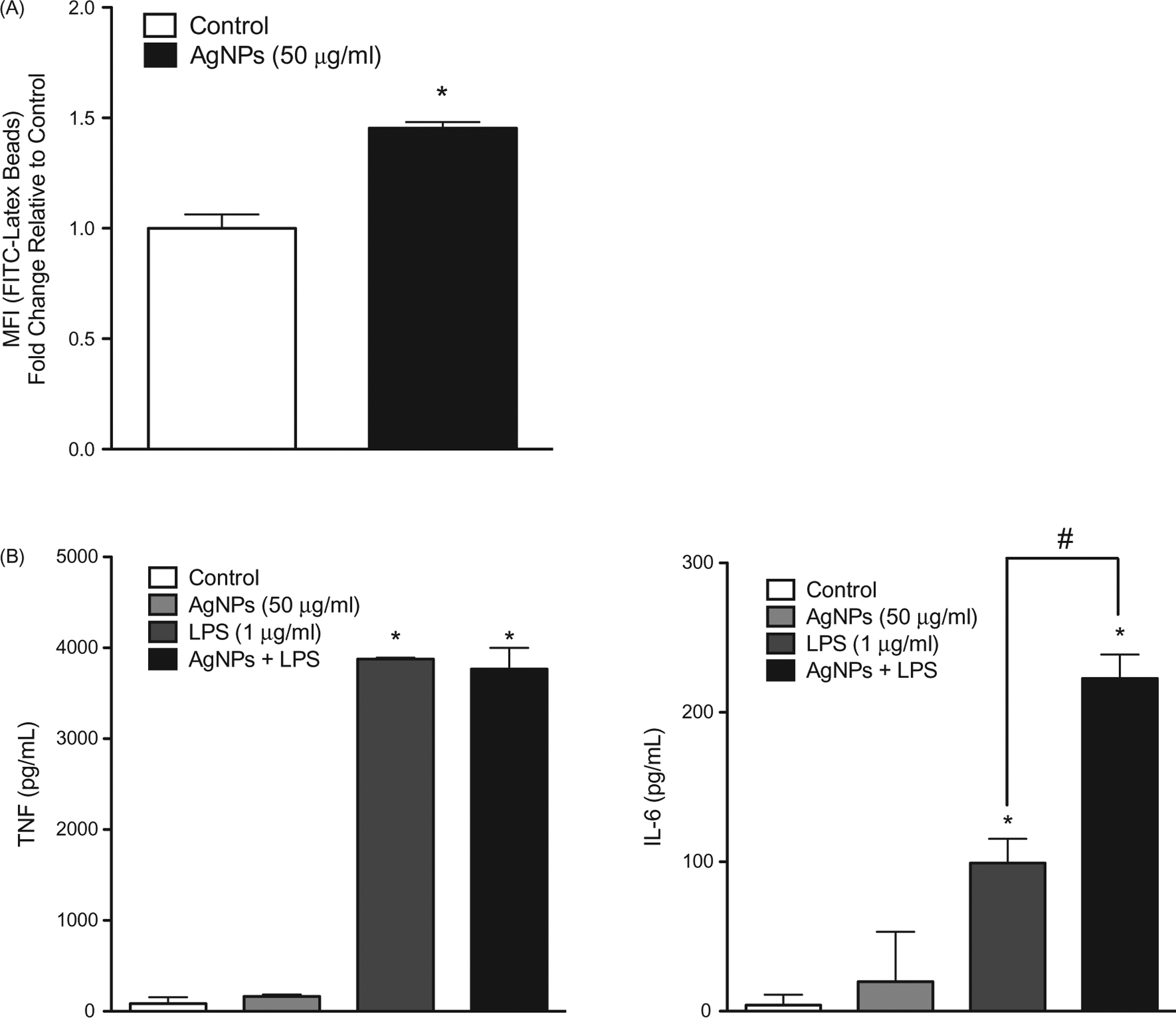
RAW 264.7 cell phagocytic ability as well as response to LPS following exposure. RAW 264.7 cells were exposed to the AgNP (50 μg/ml) for 24 h. After washing, the cells were then (A) exposed to latex beads coated with fluorescent-rabbit IgG for 4 h and uptake of beads were then measured in a flow cytometer or (B) challenged with 1 ng LPS/ml for 2 h and release of TNFα (left panel) and IL-6 (right panel) was subsequently evaluated (ELISA). Values shown are means ± SEM (*N* ≥ 3). *p < 0.05 vs. control. ^#^*p* < 0.05 vs. LPS-only group.

**Figure 6.**
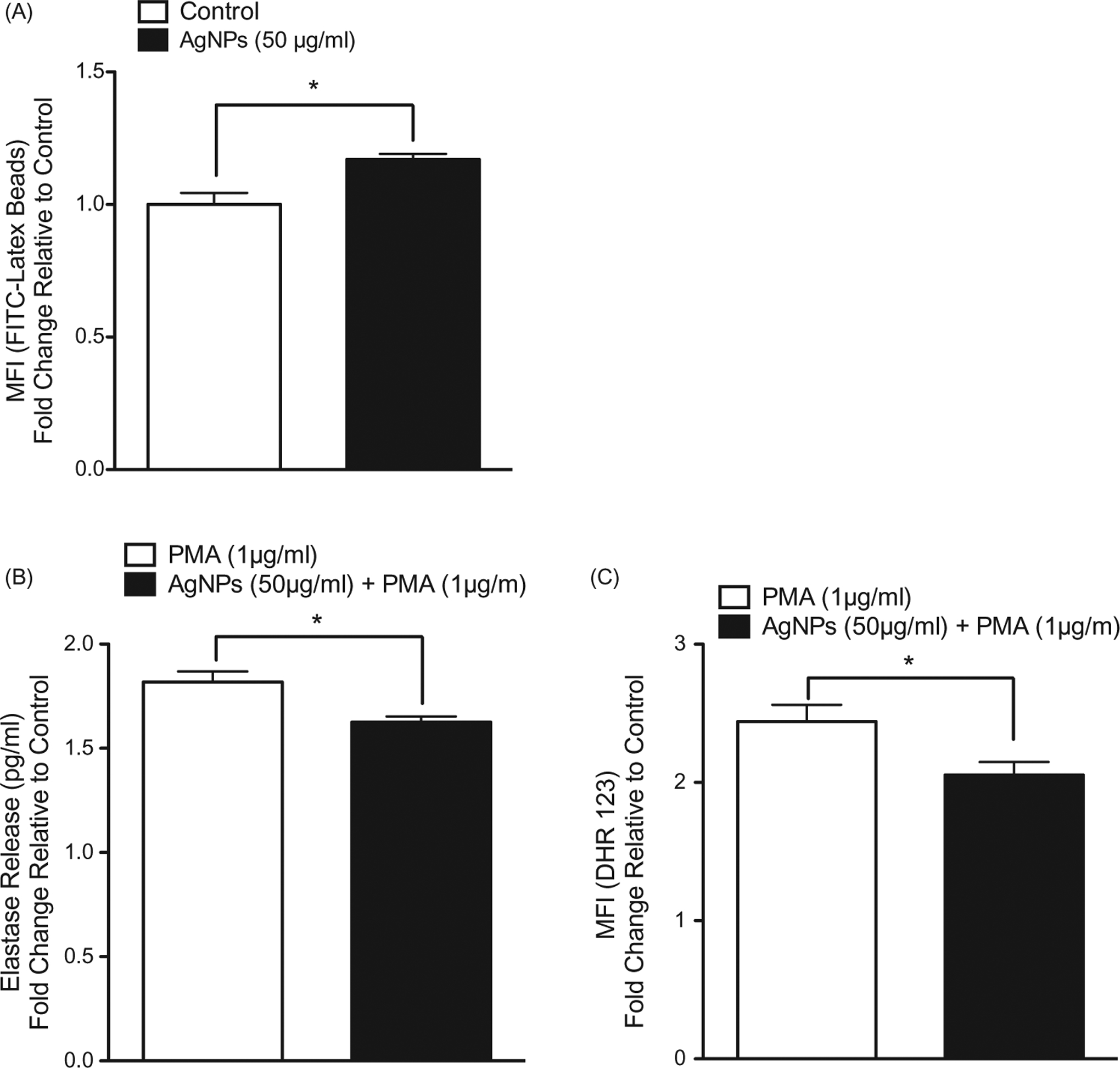
MPRO 2.1 cell phagocytic ability as well as response to PMA following exposure. MPRO 2.1 cells were exposed to the AgNP (50 μg/ml) for 24 h. After washing, the cells were then (A) exposed to latex beads coated with fluorescent-rabbit IgG for 4 h and uptake of beads was then measured in a flow cytometer, (B) challenged with 1 μg PMA/ml for 1 h after which neutrophil elastase release was measured (ELISA), or (C) challenged with PMA (1 μg/ml) for 15 min after which oxidative burst was subsequently evaluated (using fluorescent DHR 123 probe and flow cytometer). Values shown are means ± SEM (*N* ≥ 3). *p < 0.05 vs. control.

The results showed that pre-exposure of RAW 264.7 cells to the test AgNP resulted in enhanced cell phagocytic ability (Figure 5(A)). Further, the pre-exposure enhanced IL-6 - but not TNFα - release in response to an LPS challenge (1 ng/ml; Figure 5(B)). In MPRO 2.1 cells, pre-exposure resulted in increased (albeit to a lesser extent than in RAW 264.7 cells) phagocytic ability (Figure 6(A)). Measures of oxidative burst (NADPH-oxidase) and degranulation of primary granules (based on elastase release) in response to PMA (1 μg/ml) showed that pre-exposure to the AgNP (50 μg/ml) for 24 h resulted in reduced cell oxidative burst (Figure 6(B)) and degranulation in response to PMA (at 15 and 60 min, respectively, Figure 6(C)).

## Discussion

Advances in the nanosciences over the past several decades have led to engineering nanomaterials with extremely precise physicochemical properties (e.g. size, shape, surface coating, solubility, electrical conductivity, etc.) and novel applications across multiple industries including healthcare, food and medicine. A surge in the use of nano-enabled materials in consumer and medical products in day-to-day life has undoubtedly resulted in increased human exposure to ENM (Vance et al. 2015). Therefore, addressing the human safety of ENM - particularly those that are commonly utilized including silver nanoparticles (AgNPs) - is of critical importance. Although there have been previous efforts that assessed the safety of commonly utilized ENM, including specific programs and consortiums by the National Institute of Environmental Health Sciences (NIEHS) as an example, inconsistency and inter-laboratory variability remains a grand challenge (Bonner et al. 2013; Schug et al. 2013; Xia et al. 2013. The current study investigated basic cellular responses to AgNP exposure including generation of ROS, oxidative stress, inflammatory response, and function in two key innate effector immune cell models, RAW 264.7 macrophages and all-trans retinoic acid (ATRA)-differentiated MPRO 2.1 promyelocyte/neutrophils. Overall, the findings described in this study demonstrated that exposure to AgNP has the potential to influence basic innate immune cell responses in the absence of direct cytotoxicity.

The mononuclear phagocytic system (MPS; also known as the reticuloendothelial system, RES) plays a crucial role in the clearance of ENM (Gustafson et al. 2015). The MPS consists primarily of tissue-resident macrophages found mainly in the liver and spleen but also in other secondary lymphatic organs. Therefore, macrophages have been the most widely utilized *in vitro* model to study the toxicity of AgNPs; however, there have been major inconsistencies between previous reports regarding the type and magnitude of activation and/or toxicity (Carlson et al. 2008; Yen et al. 2009; Park et al. 2010; 2011a; Shavandi et al. 2011; Hayashi et al. 2012; Lim et al. 2012; Arai et al. 2015; Giovanni et al. 2015). Nevertheless, emerging evidence indicates that the toxicity of AgNPs in macrophages is potentially driven by ROS generation leading to oxidative damage and activation of inflammatory responses eventually resulting in cell death (Yen et al. 2009; Park et al. 2010; Park et al. 2011a).

In the study here, exposure to AgNP for 24 h was not associated with reduced RAW 264.7 cell viability even at high concentrations and despite a large uptake of the nanoparticles in the 24h exposure period. Such lack of cell death following exposure, when compared to outcomes in previous studies, could be attributed to a number of reasons including preparation of the AgNP (e.g. use of basic physiological buffer vs. cell culture media, presence of serum vs. reduced serum or serum-free, type of serum, etc.), AgNP physicochemical properties (e.g. size, shape, surface functionalization, etc.), cell density, cell-particle ratio, and even sensitivity of the cell model (Teeguarden et al. 2007; Kong et al. 2011; Albanese et al. 2012; Kettler et al. 2014). Indeed, such variations in experimental conditions have been shown to drastically influence AgNP biological (toxicological) behaviors (Nel et al. 2006; Shvedova et al. 2010; Gatoo et al. 2014). Similarly, exposure to AgNP here was not associated with reduced cell viability of the MPRO 2.1 cells. These results suggested to us that exposure to the AgNP in serum-supplemented media was not associated with major cytotoxicity in key cell models associated with innate immunity.

Due to their large surface to volume ratio, ENM have been previously shown to generate ROS in most cellular models (Fu et al. 2014). In fact, this represents the current paradigm for ENM-induced cellular toxicities (Manke et al. 2013). Evidence from several immune cell models has indicated that exposure to AgNP triggers a burst of ROS formation that leads to cellular oxidative stress, macromolecular damage, and even cell death (Lappas 2015). It is noteworthy that physicochemical properties of AgNP (e.g. size, shape, surface coating, and charge, etc.), as well as experimental conditions (e.g. presence of serum in cell culture media), are critical factors in AgNP-induced ROS generation and subsequent toxicity. In the present study, exposure to the AgNP resulted in increases in ROS generation in RAW 264.7 cells that were more prominent with increasing lengths of exposure, suggesting a particle internalization-driven mechanism of ROS generation (Droge 2002). Generation of ROS was also observed in the MPRO 2.1 cells. Despite reductions in RAW 264.7 cell total glutathione levels due to the 24h exposure to the AgNP, there was a lack of cellular oxidative damage in both cell types. Such results indicated to us that such ROS generation was not overwhelming the cells anti-oxidant systems or at least up to and through 24 h of exposure (Apel and Hirt 2004). This suggested to us that exposure to AgNP involved a transient ROS generation that was not related to subsequent major oxidative damage/cell toxicity.

Due to their novel physicochemical properties and in contrast to micron-size particles, ENM cross biological barriers and have wide biodistribution and tissue accumulation. Many distal sites (e.g. liver, spleen, kidneys, lungs, brain, testes, etc.) are impacted as a result of drainage through lymphatics (Manolova et al. 2008; Kettiger et al. 2013). With such a wide distribution in a body, there is a concern for not only ENM-mediated interactions with blood cells and the endothelium but also potential direct interactions with tissue-resident immune cells, including macrophages. Optimal function of tissue-resident innate effector immune cells is of critical importance to the overall function of the immune system (Kumar et al. 2011; Wernersson and Pejler 2014).

Although the majority of work in evaluating ENM-induced immunomodulation has focused on assessing direct cytotoxicity to immune cells and pro-inflammatory responses, any absence of such manifestations does not necessarily indicate a lack of adverse cellular responses among immune cells. Indeed, the ability of cells to respond properly to environmental stressors (pathogens, particulates, radiation, etc.) following exposure to ENM is of critical importance. Most importantly, emerging literature has demonstrated the potential of ENM (including metal and metal-oxide nanoparticles) to alter innate immune cell functions (covering activation and/or suppression) at sub-cytotoxic concentrations (Comfort et al. 2011; Andersson-Willman et al. 2012; Seydoux et al. 2014). Furthermore, it has been shown in *in vivo* settings that exposure to AgNP influences immune cell functions in response to immunologic stimuli (de Jong et al. 2013; Vandebriel et al. 2014). For instance, AgNP exposure of rats resulted in suppression of their natural killer cell activities and altered *ex vivo* cytokine/chemokine release in response to Concanavalin A (ConA)- and lipopolysaccharide (LPS)-mediated activation of their spleen cells.

Although the data from the current study showed a lack of direct cellular toxicity in response to AgNP exposure, the possibility of modulating normal cell function and responses to known immunological stimulation cannot be outright excluded. To gain some insights into whether AgNPs could modulate immune function, the cells were exposed to the test AgNP for 24 h and then assessed for key functions in response to known immunologic stimuli. Interestingly, the results here showed that exposure to AgNP resulted in enhanced cell phagocytic ability in both cell lines. Similar findings have been shown in THP-1 cells (human monocytic line) in response to an exposure to a number of metal oxides nanoparticles (DeLoid et al. 2016). However, it has also been reported in bone marrow-derived macrophages that pre-exposure to amorphous silica and super-paramagnetic iron oxide nanoparticles (SPION) for 24 h led to reduced phagocytic abilities of the exposed cells. The current study also showed that exposure to AgNPs might be associated with the modulation of inflammatory responses in RAW 264.7 cells subsequently treated with LPS. Modulation of responses to LPS was previously noted in macrophages following pre-exposure to amorphous silica and SPIONs for 24 h (Kodali et al. 2013).

Although pre-exposure to the AgNP enhanced the phagocytic ability of MPRO 2.1 cells, it resulted in a reduction of PMA-induced cell degranulation of primary granules as well as of the cellular oxidative burst. To our knowledge, this is the first report to show the potential of AgNP to modulate neutrophil functions/responses to a known immunological stimulant without exerting direct (cyto)toxicity. A few earlier reports investigated multiple endpoints of toxicity (e.g. release of pro-inflammatory cytokines, neutrophil extracellular traps, cell death, etc.) in response to AgNP exposure in primary neutrophils (Poirier et al. 2014, 2016; Soares et al. 2016). However, those studies never attempted to study the consequences of exposure to sub-(cyto)toxic concentrations of the AgNPs. Interestingly, a recent report assessed the influence of AgNP on different neutrophil subpopulations isolated from healthy volunteers (Fraser et al. 2018). The study found that exposure to AgNP was neither associated with classical pro-inflammatory responses nor direct cell toxicity as previously reported by others. However, this study did show that the exposure resulted in the maturation of immature neutrophils and activation of mature neutrophils. As was the case in the present study, such findings provided further evidence of AgNP immunomodulatory properties in the absence of major cellular cytotoxicities.

## Conclusion

Emerging evidence demonstrates the potential immunomodulatory properties of metal and metal oxide ENM (Alsaleh and Brown 2018). Furthermore, and in accordance with the current findings, such evidence indicates that exposure to ENM of the same composition and physicochemical properties may result in activation or suppression of cellular pathways and functions (Kodali et al. 2013; DeLoid et al. 2016). Although the exact molecular mechanisms are still lacking, one report suggested a potential mechanism through interaction with the scavenger receptor type A (SR-A) (Kodali et al. 2013). In addition, it has been shown that internalization of ENM resulted in reprogramming of genes involved in multiple cellular processes, including cell differentiation, adhesion, inflammation, and immune responses (Kodali et al. 2013). Therefore, understanding ENM-immune cell interactions at the molecular level – beyond direct cellular toxicity - is of crucial importance for the future development of ENM in nanomedicine. By this, investigators might utilize a safety-by-design approach and thereby exploit ENM properties toward desired clinical outcomes, including immune suppression in graft transplantation, immune activation in cancer therapy, etc. (Dobrovolskaia et al. 2016).

## Supplementary Material

Supp1

## Figures and Tables

**Table 1.** Characterization of test AgNP in DI water and cell culture media.

Vehicle	Hydrodynamic size (nm)	Zeta potential (mV)	PDI
Dl Water	30.08 ± 0.29	−35.03 ± 1.65	0.27 ± 0.02
DMEM	277.27 ± 0.01	−10.97 ± 0.50	0.21 ± 0.01
IMDM	283.57 ± 0.01	−16.27 ± 0.47	0.20 ± 0.01
