# Supplementary figures and images for: Silver nanoparticle immunomodulatory potential in absence of direct cytotoxicity in RAW 264.7 macrophages and MPRO 2.1 neutrophils
Source: J Immunotoxicol. Author manuscript; Available in PMC 2020 Dec 1. (PMC7135879; doi:10.1080/1547691X.2019.1588928)

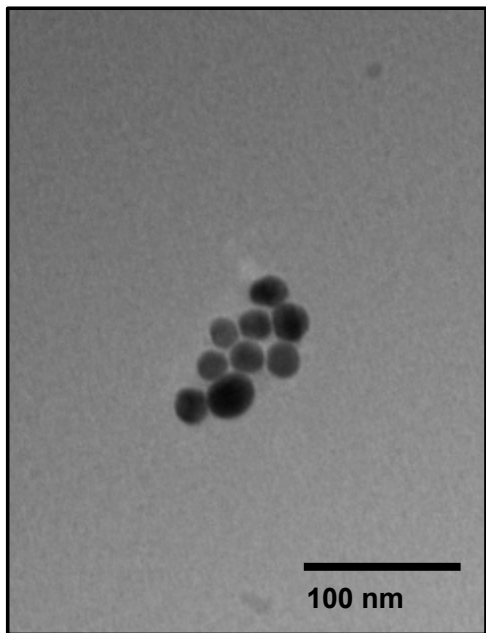

**Figure S1**

**A**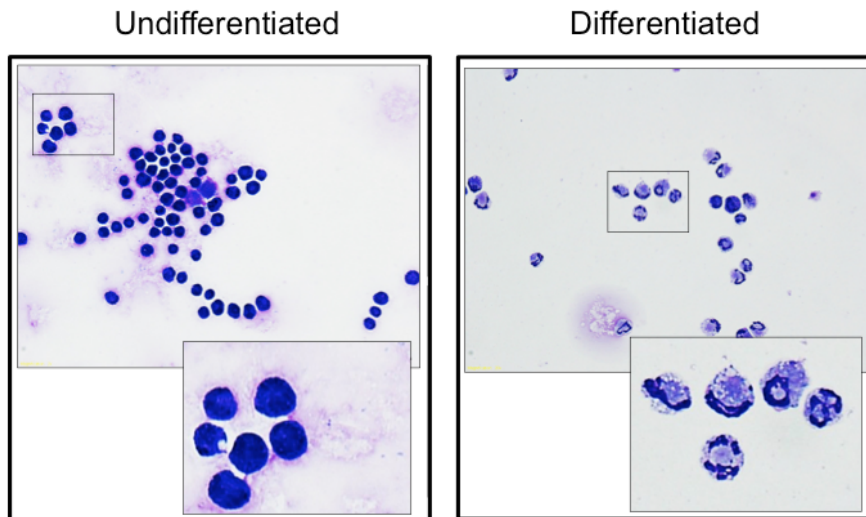**B**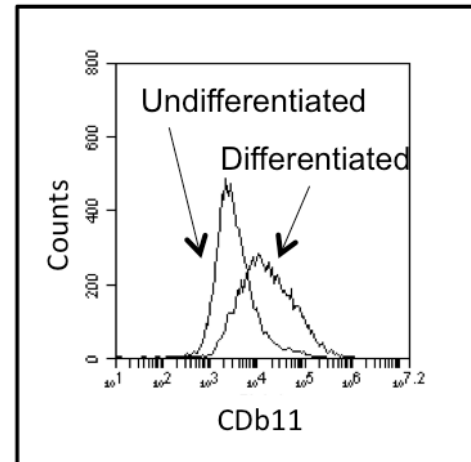**Figure S2**

**A**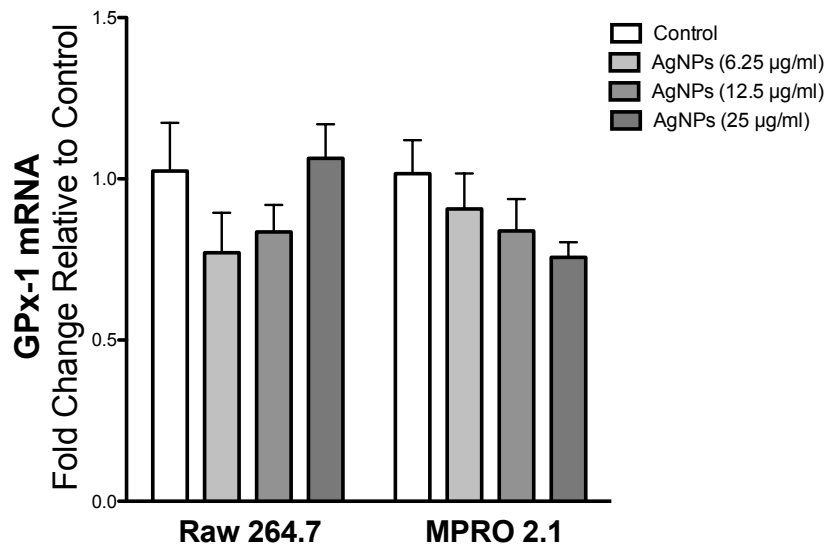**B**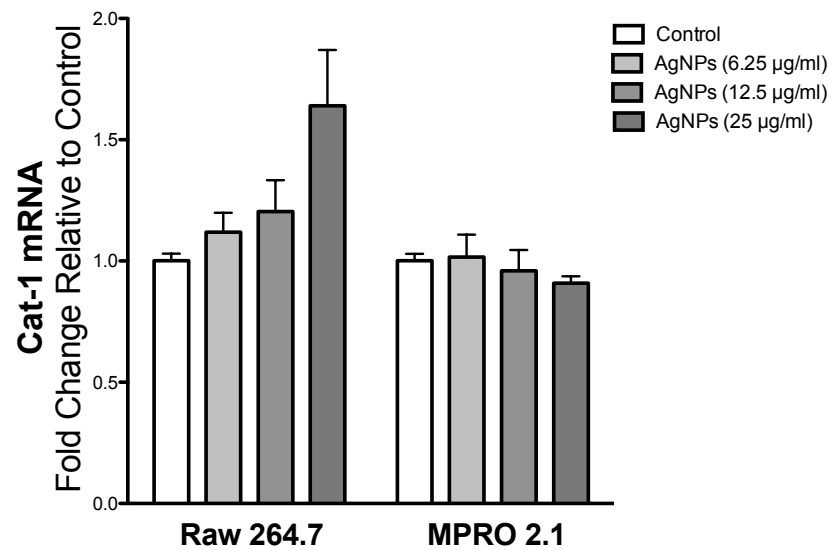**Figure S3**

## RAW 264.7

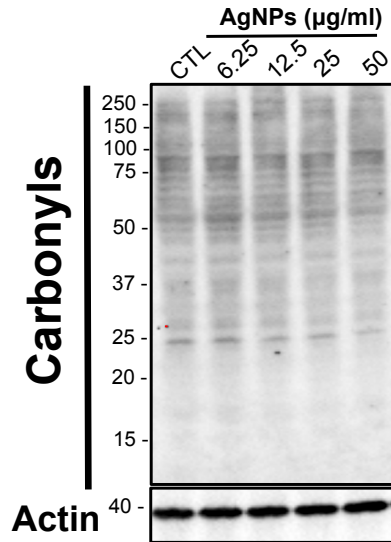

## MPRO 2.1

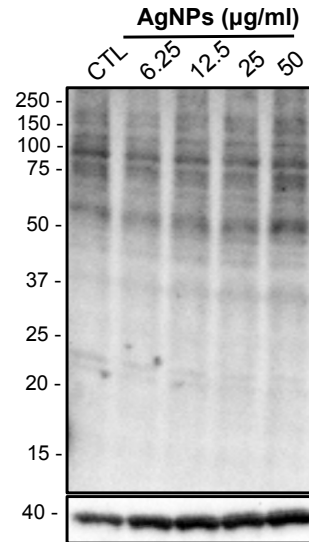

**Figure S4**

Supplement: Supp1 [file NIHMS1572165-supplement-Supp1.pdf]
